# Magnitude and predictors of poor glycemic control in patients with diabetes at Jimma Medical Center, Ethiopia

**DOI:** 10.1038/s41598-023-42774-y

**Published:** 2023-09-24

**Authors:** Mariam Dubale, Kaleab Gizaw, Dula Dessalegn

**Affiliations:** 1 Department of Clinical Pharmacy, School of Pharmacy, Jimma University, Jimma, Oromia Ethiopia; 2 Department of Pharmacology, School of Pharmacy, Jimma University, Jimma, Oromia Ethiopia; 3 Department of Clinical Pharmacy, School of Pharmacy, Jimma University, Jimma, Oromia Ethiopia; 4 Department of Quantitative Pharmacology, Merck KGaA, Darmstadt, Hessen Germany

**Keywords:** Endocrinology, Health care

## Abstract

Despite the development of new medications over the past decade to aid in the control of blood glucose, most diabetic patients often do not reach recommended glycemic targets of glycated hemoglobin (HbA1C) < 7% in daily clinical practice because of many contributing factors. This study was designed to assess the magnitude and predictors of poor glycemic control among adult diabetic patients on ambulatory chronic care follow-up at Jimma Medical Center. A cross sectional study was conducted on 307 adult diabetic patients between January 2 and April 30, 2022. Representative samples were selected using a systematic random sampling technique. Predictors of poor glycemic control were assessed using a binary and multi variable logistic regression. Data analysis was performed using Statistical Package for Social Science version 25 and R in the R studio environment. A total of 307 adult diabetic patients were included in the study making a response rate of 93%. Out of 307 adult diabetic patients, majority (62.5%) were males. Mean age of the patients was 48.91 ± 15.68 years. The majority, 221 (72%), of patients had poor glycemic control. Non-adherence of patients to medications (AOR = 3.36, 95% CI 1.16–9.72, *p* = 0.04), no formal education (AOR = 3.84, 95% CI (1.06–13.93, *p *= 0.04), therapeutic inertia (AOR = 3.16, 95% CI 1.61–6.20, *p* = 0.001) and poor diabetic knowledge (AOR = 4.79, 95% CI 1.56–14.68, *p* = 0.006) were found to be independent predictors of poor glycemic control. Nearly three fourth of diabetic patients in the present study had poor glycemic control and were at higher risk of developing diabetic complications or already developed it. These results highlight the need for appropriate management of patients focusing on adherence to medications, education, therapeutic inertia and diabetic knowledge to maintain good glycemic control and improve adverse outcomes of the disease in this study setting.

## Introduction

Diabetes Mellitus (DM) refers to a group of common metabolic disorders that share the phenotype of hyperglycemia where the hyperglycemia can be due to defects in insulin secretion, insulin actions or both^1^.

Globally, around 463 million people were living with diabetes in 2019. The prevalence of DM among adult Africans aged between 20 and 79 is 3.9%. Ethiopia also faced an increasing rate of DM among its population being the third country in Africa in terms of DM burden national prevalence of 3.2%^2,3^.

DM is estimated to be associated with 11.3% of global deaths from all causes among adults where almost half of deaths are in the working age group with African region having the highest estimate. The economic impact of diabetes is expected to continue to grow. It is projected that expenditure will reach 845 billion United States Dollar by 2045^2^.

Current practice guidelines recommend lifestyle and dietary modifications, usually followed by metformin monotherapy and the further addition of other therapies, including oral and injectable medications if the Glycated Hemoglobin A1C (HbA1C) target is not achieved after approximately 3 months for type 2 diabetes mellitus (T2DM). While for type 1 diabetes (T1DM), insulin is the only drug of choice in addition to lifestyle modification^4^.

According to American Diabetes Association (ADA), if a patient presents with HbA1C of 9%, it is recommended that the patient will require dual combination therapy to achieve the target A1C level. The choice of which agent to add is based on drug-specific effects and patient factors^5^.

Many non-pregnant adult patients with diabetes are expected to achieve a glycemic goal of A1C < 7% while on treatment but glycemic management can be individualized based on patient characteristics^6,7^.

Despite the development of new medications over the past decade to aid in the control of blood glucose, most diabetic patients often do not reach recommended glycemic targets of A1C < 7% in daily clinical practice^8^. A recent meta-analysis of 16 studies conducted in Ethiopia showed 66.8% of patients not achieving good glycemic control based on HbA1C measurements^9^. Poor and inadequate glycemic control among diabetic patients constitutes to be a major public health problem and major risk factor for the development of diabetes complications^8^.

Many factors are associated with poor glycemic control. Few factors include comorbidity, poor adherence to treatment, diet, and exercise in addition to various sociodemographic and clinical factors^10,11^.

One of the most common yet not well studied factors is failure to intensify treatment despite sub optimal glycemic control^10^. Such a delay in treatment intensification is termed as Therapeutic inertia (TI)^12^.

Optimal management of hyperglycemia reduces the risk of complication dramatically. For instance, results from many observational studies have revealed that lowering HbA1C concentrations from 9.1% to 7.3% reduces the risk of macrovascular disease by 41%, retinopathy by 63%, neuropathy by 60% and nephropathy by 54%. On the contrary, every increase in HbA1C can increase the cardiovascular and microvascular event rates by up to 18% and 30% respectively^13^.

Although many studies were conducted on factors contributing to poor glycemic control, almost all these studies focused on patient related factors giving less or no emphasis on health care professional related factors such as therapeutic inertia. Therefore, this study was conducted to assess magnitude of poor glycemic control and its predictors with a focus on therapeutic inertia*.*

## Methods

The study was carried out at Jimma Medical Center (JMC), the largest general Public University Hospital in Southwest Ethiopia. A hospital based cross-sectional descriptive study was conducted between January 2-April30, 2022. The study populations were all adult diabetic patients attending the chronic care follow up unit of JMC who fulfilled the eligibility criteria.

### Eligibility criteria

#### Inclusion criteria

- Age ≥ 18 years old
- Adult diabetic patients who spent at least 3 months on medication

### Exclusion criteria

- Patients with Iron deficiency anemia (IDA) (because IDA falsely elevates the level of A1C)
- Patients with a history of recent blood transfusion (≤ 3 months) (because it falsely increases the A1C test results).
- Patients with chronic kidney (CKD) (because complications of CKD such as malnutrition and anemia can affect the A1C test results)
- Patients with gestational diabetes
- Hospitalized patients and/or patients with psychiatric disorder during the data collection time (since there was a diabetes self-care practices assessment)

### Sample size determination and sampling technique

The sample size was calculated based on the following assumptions: use of a population survey/descriptive study formula with a 95% confidence level; population size 3058 (total number of adult diabetic patients having a regular follow up at JMC): Expected frequency 59.5% (based on a cross sectional study conducted among adult diabetic patients in JMC using HbA1C)^14^; Design effect 1; Clusters 1. The final sample size, as calculated using Epi-Info Version.7.2.5 statistical software for medical research studies (Atlanta, Georgia, USA), was 330. By dividing the total number of adult diabetic patients having a regular follow up at JMC by the sample size, we obtained Kth interval of 9. Accordingly, every ninth person aged ≥ 18 years was included in the study. Thus, a total of 330 study subjects were selected through a systematic random sampling procedure.

### Data collection tool

Data regarding sociodemographic characteristics, medication adherence, diabetic self-care activities, diabetic knowledge and behavioral related variables were collected by interviewing patients face to face using a semi- structured questionnaire.

Therapeutic inertia (TI) was assessed based on data obtained from patients’ chart which includes information about the medication the patient was taking, its dose, frequency and duration and the obtained information was compared against the ADA guideline to look for the presence or absence of TI^4^.

Morisky’s 8 item medication adherence questionnaire was used to assess adherence to medication^15^. Accordingly, good adherence to medication is considered if the patient score > 6, and poor medication adherence if score is ≤ 6.

Summary of diabetes self-care activities (SDSCA) scale which contains 10 questions about four domains; diet, exercises, blood sugar test and foot care was used to assess behavioral factors after minor changes were made to suit the present study^16^. For all domains frequency of self-care activity in the last 7 days were measured. Based on the overall mean score, it was classified as having good self-care practice if the patient scored ≥ 4 or poor self-care practices if the patient scored < 4^16^.

Diabetes knowledge was assessed using diabetes knowledge questionnaire (DKQ)^17^. DKQ consists of 24 questions and helps to estimate general patient knowledge of diabetes. The score for each participant was determined by dividing the number of correct answers by the total number of questions. Patients’ overall level of knowledge was grouped into three on the basis of their DKQ scores: as good, acceptable and poor knowledge if their overall scores are > 75%, 61–75%, and ≤ 60%, respectively.

The data collection tool was first prepared in English and then translated to Amharic and Afaan Oromo. Finally, it was translated back to English to ensure validity of translation.

### Laboratory test

HbA1C was used to measure the status of glycemic control. Two laboratory technicians of Jimma medical center were involved in drawing blood and performing tests. Accordingly, about 2 ml of whole blood was collected from a single venipuncture of diabetic patients using an EDTA vacutainer tube for the determination of HbA1C. HbA1C levels were measured using Cobas 6000 Clinical Chemistry analyzer (Roche Diagnostics GmbH, Mannheim, Germany).

### Data management and quality assurance

To ensure data quality, reliability of data extraction forms was checked by doing pretest on 5% of the sample size. Training was given for data collectors, and they were supervised by the principal investigator. Accuracy and completeness of data was checked daily after data collection time.

### Data processing and statistical analysis

Data was entered, coded, and cleared using Epi data version 4.6 software. Data entry, processing and analysis were performed using Statistical Package for Social Science (SPSS) version 25.0 (SPSS Inc., Chicago, Illinois, USA) and R software version 4.2.2 in Rstudio Environment (R Core Team (2022). R: A language and environment for statistical computing. R Foundation for Statistical Computing, Vienna, Austria. URL https://www.R-project.org/) with α = 0.05. Plots were made using the ggplot2 package in RStudio environment (Wickham. ggplot2: Elegant Graphics for Data Analysis. Springer-Verlag New York, 2016.)

Data was expressed as mean ± SD for continuous variables and as frequencies and percentages for categorical variables.

Chi-square test was used to assess the associations between categorical variables. A multivariable logistic regression analysis was used to assess predictors of glycemic control. All variables were initially tested for an association with glycemic control in bivariate. Those variables demonstrating a bivariate association with at least marginal significance of (*P* < 0.25) were included in a multivariable model. A stepwise back ward elimination method was used to identify the independent predictors of poor glycemic control. Hosmer and Lemeshow’s test of goodness fit was used to test model fitness. Adjusted odds ratio was used to measure strength of association.

A *p*-value of < 0.05 (with 95% confidence interval) was considered statistically significant. Finally, the result was presented using narrative, tables, graphs, and charts.

### Ethical clearance

Prior to data collection, ethical clearance was obtained from institutional review board (IRB) of Jimma University, College of Health Sciences (RefNo.IRB000253/2022). Verbal consent from participants was obtained before the interview. In addition, a written informed consent was granted from study participants prior to initiation of the study. To keep confidentiality of patient information, each patient data was coded, and only patient initials were used. The objective of study was made clear to concerned bodies including Jimma Medical Center. The study was conducted in accordance with the Declaration of Helsinki.

### Ethics approval and consent to participate

Prior to data collection, ethical clearance was obtained from institutional review board (IRB) of Jimma University, College of Health Sciences (RefNo.IRB000253/2022). The study was performed in accordance with good clinical practice.

Before the commencement of the study, study participants were informed about the nature and objectives of the study. They were given adequate information and were invited to be part of the research. They were not required to decide on the first day to participate in the research or not. Before they can decide, they were allowed to talk to anyone they felt comfortable with about the research.

If there were some words that they did not understand, they were allowed to ask, and data collectors promised take time to explain. If they had questions later, they were allowed to ask investigators, the study doctor, or the staff.

Regarding the glycated hemoglobin test, they were notified that the data collectors will take 2 ml of blood from their arm using a syringe and needle. After the test, they were assured that any leftover blood sample would be destroyed.

In addition, patients were notified that their participation in the research was entirely voluntary. They were given a chance to choose whether to participate or not. Whether they choose to participate or not, all the services they receive at this clinic will continue and nothing will change. If they choose not to participate in the research, we told them that they will be offered the treatment that is routinely offered in chronic care unit of Jimma Medical Center.

They were also informed that they can change their mind later and stop participating even if they agreed earlier. The information that they provide will be kept confidential in that the information about them that was collected during the research will be put away and no-one, but the researchers will be able to see it.

They were also informed that the knowledge that the researchers get from doing the research will be shared with them through community meetings before it was made widely available to the public. After these meetings, they were notified and agreed that the results will be published in order that other interested people may learn from the research.

After a brief explanation of the objective and nature of the study, both verbal and written consent was obtained before prior to initiation of the study.

## Results

### Socio-demographic characteristics of study participants

A total of 307 adult diabetic patients were included in the study making the response rate of 93%. There were 192 (62.5%) male participants. Majority of the study participants were in the age range of 36 to 55 years, accounting for about 42% of the total patients (n = 129). The mean (± SD) age of the study participants was 48.91 ± 15.68 with the minimum age of 18 and maximum of 90.

Of all study participants, 188 (61.2%) were Oromo and 171(55.7%) were Muslim. Majority of them were married 224 (73%) and rural dwellers 157 (51.1%). About 38.4% (n = 118) of the participants had primary education. From the total of 307 patients, majorities were merchants 76 (24.8%) and most of them had no regular income 117(38.1%) (Table1).

**Table 1:** Socio-demographic characteristics of adult diabetic patients on follow up at Jimma Medical Center, Southwest Ethiopia, 2022 (n = 307).

Patient characteristics		n	(%)
Sex	Male	192	62.5
	Female	115	37.5
Age	18–35	62	20.2
	36–45	129	42
	> 45	116	37.8
Ethnicity	Oromo	191	62.2
	Amhara	60	19.5
	Kaffa	40	13
	Gambella	16	5.3
Religion	Muslim	176	57.3
	Orthodox	90	29.3
	Protestant	41	13.4
Marital status	Married	224	73
	Single	44	14.3
	Widow	28	9.1
	Divorced	11	3.6
Occupational status	Merchant	76	24.8
	Farmer	72	23.5
	Government employee	54	17.6
	Housewife	32	10.4
	Retired	28	9.1
	Student	25	8.1
	Daily labor	20	6.5
Place of residence	Rural	157	51.1
	Urban	150	48.9
Level of education	No formal education	87	28.3
	Primary education	118	38.4
	Secondary education	63	20.5
	Higher education	39	12.7
Monthly income in ETB^a^	No regular income	117	38.1
	500–1000	45	14.7
	1001–2000	57	18.6
	> 2000	88	28.7

^a^*ETB* Ethiopian Birr.

### Social drug use and behavioral factors

Few study participants were current users of social drugs where the most used substances were Khat (32.2%) and alcohol (3.0%). Majority of study participants were non-alcoholic and only 3% of the participants were current alcoholics. There were no current smokers and majority of the participants were non-smokers. In Addition, few respondents were current consumers of traditional medicine (13, 4.2%) (Table 2).

**Table 2:** Social drug use and behavioral factors among adult diabetic patients on follow up at Jimma Medical Center, Southwest Ethiopia, 2022 (n = 307).

Social drug use and behavioral factors		n	(%)
Khat chewing	Yes	99	32.2
	No	208	67.8
Alcohol	Current alcoholic	9	3
	Ex-alcoholic	98	31.9
	Non alcoholic	200	65.1
Smoking	Current smoker	–	–
	Ex-smoker	14	4.6
	Non smoker	293	95.4
Traditional medicine	Yes	13	4.2
	No	294	95.8
Adherence to medications	Poor	220	71.7
	Good	87	28.3

According to Morisky’s 8 item medication adherence scale, most patients (220, 71.7%) had poor adherence to their anti-diabetic medications.

### Clinical information of study participants

Most of the study participants had type 2 diabetes mellitus 251, (81.8%). One hundred thirty-two (43.0%) patients had diabetes duration of more than ten years. The mean duration of diabetes mellitus was 5.18 ± 4.21, with the smallest duration of 3 months and the largest duration being 18 years. More than half (51.1%) of the study participants had one or more comorbid conditions with hypertension being the 121 (77%) most prevalent comorbid condition. Most participants 159 (51.8%) were not aware of their family history of diabetes. About 20.8% of study participants had one or more diabetic complication and the most prevalent diabetic complication was neuropathy accounting for 76.6% (n = 49). Most participants visited the chronic care unit every month 201 (65.5%) and most of them 201(65.5%) had no missed follow up (Table 3).

**Table 3:** Clinical information of adult diabetic patients on follow up at Jimma Medical center, Southwest Ethiopia, 2022 (n = 307).

Clinical information		n	(%)
Type of DM^a^	Type 1 DM	56	18.2
	Type 2 DM	251	81.8
Duration of DM (in years)	< 5 years	78	25.4
	5–10 years	97	31.6
	> 10 years	132	43
Family history of DM	Yes	66	21.5
	No	82	26.7
	I do not know	159	51.8
Comorbid condition	Yes	157	51.1
	No	150	48.9
Number of co-morbid condition	1	141	89.8
	2	15	9.5
	> 2	1	0.7
Common co-morbidities	HTN^b^	121	77.1
	HTN + CHF^c^	22	14
	CHF	14	8.9
Diabetic complications	Present	64	20.8
	Absent	243	79.2
Common diabetic complications	Neuropathy	52	81.3
	Retinopathy	8	12.5
	Nephropathy	4	6.2
Number of visits per year	6 times	106	34.5
	12 times	201	65.5
Number of missed follow up per visit	0	201	65.5
	01-3	102	33.2
	> 3	4	1.3

^a^*DM* Diabetes Mellitus.

^b^*HTN* Hypertension.

^c^*CHF* Congestive Heart Failure.

### Treatment related information

All diabetic patients in the present study were on one or multiple anti-diabetic medications. Most patients 161 (52.4%), however were on monotherapy with insulin being the most common 109 (35.5%) patients. Most of study participants 132 (43.0%) stayed on medication for more than 10 years. Among 157 patients with comorbid conditions, 155 (98.7%) patients were on concomitant therapy. Of these, 45.2% of them were on Enalapril and acetyl salicylic acid combination therapy (Table 4).

**Table 4:** Types of antidiabetic medications among adult diabetic patients on follow up at Jimma Medical center, Southwest Ethiopia, 2022 (n = 307).

Treatment related information		n	(%)
Duration of therapy (in years)	< 5	78	25.4
	5–10	97	31.6
	> 10	132	43
Medications	Monotherapy	156	50.8
	Combination therapy	151	49.2
Type of antidiabetic medications	Insulin	109	35.5
	Metformin + Insulin	87	28.3
	Metformin + Glibenclamide	64	20.8
	Metformin	47	15.3
Concomitant medications	Yes	155	50.5
	No	152	49.5
Type of concomitant medication	Enalapril + ASA^a^	70	45.2
	Enalapril + ASA + hydrochlorothiazide	49	31.6
	Enalapril + atenolol + ASA	20	12.9
	Enalapril + atorvastatin	16	10.3

^a^*ASA* Acetyl Salicylic Acid.

### Diabetic knowledge and self-care activities

**Figure 1:**
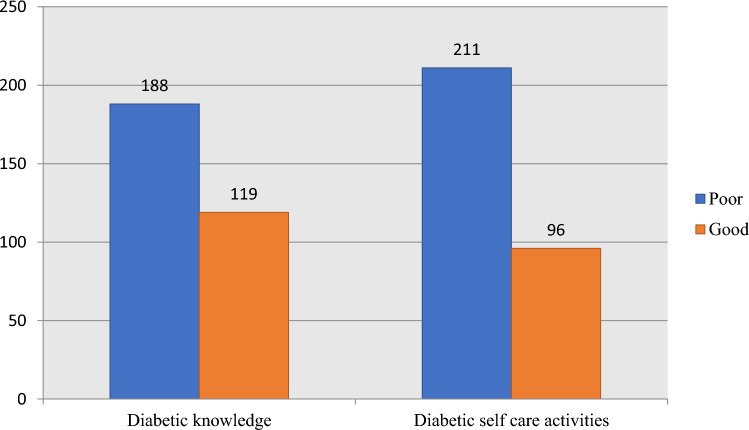
Diabetic knowledge and Diabetic Self-care Activity Profile of Adult Diabetic Patients on Follow up at JMC, Southwest Ethiopia, 2022 (n = 307).

Most of the patients had poor knowledge about diabetes 188 (61.2%). The mean score of diabetic knowledge among study participants was 13.11 ± 3.89 (54.62%) with a minimum and maximum of 6 and 21 respectively. In addition, majority of the patients 211 (68.7%) had poor diabetic self-care practice. The mean score of diabetic self-care among the study participants was 3.50 ± 1.50 (minimum 1 and maximum 7) (Fig. 1).

### Magnitude of poor glycemic control

**Figure 2:**
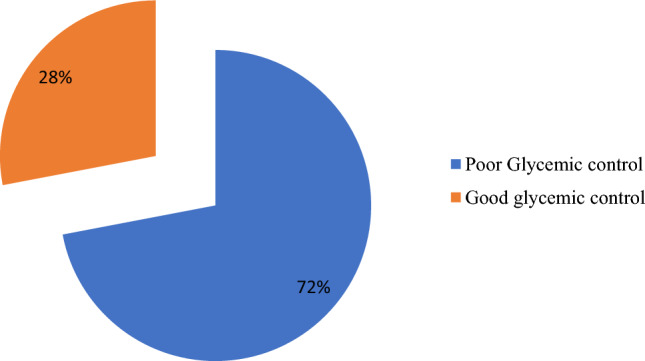
Status of Glycemic Control among Adult Diabetic Patients on Follow up at Jimma Medical Center, Southwest Ethiopia 2022 (n = 307).

Among the study participants, nearly three fourths 221 (72%) of them had poor glycemic control. The mean HbA1C of study participants was 8.97 ± 2.74 (3.45–16.80). The magnitude of poor glycemic control was higher in patients with type 2 diabetes mellitus (73.70%) and patients with comorbid conditions (51.60%). In contrary, a higher percent of poor glycemic control was observed in diabetic patients who don’t have diabetic complications 173 (78.3%) when compared to patients with diabetic complications (Fig. 2).

### Magnitude of therapeutic inertia

**Figure 3:**
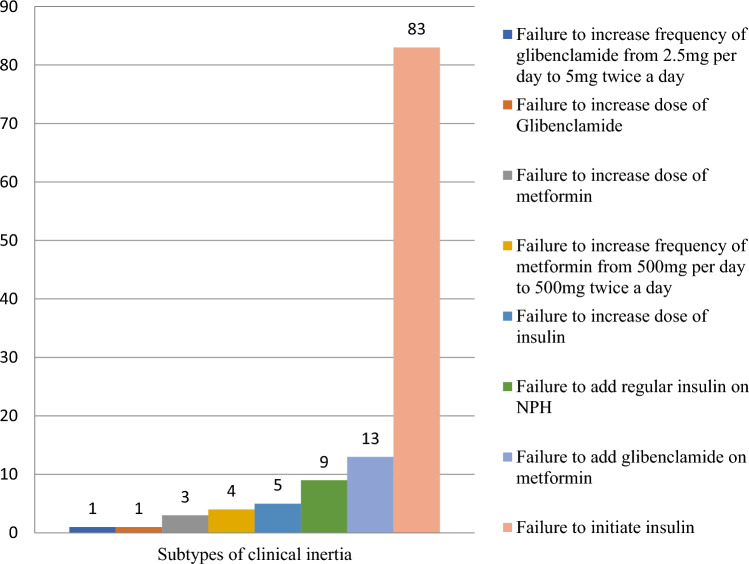
Subtypes of Therapeutic Inertia among Adult Diabetic Patients on Follow up at Jimma Medical Center, Southwest Ethiopia, 2022 (n = 119).

TI was assessed for patients who had their blood glucose uncontrolled. In this manner, among 221 patients who had their blood glucose uncontrolled, 119 patients (53.8%) were found to have TI. Accordingly, the types of therapeutic inertia observed in the present study were failure to initiate insulin 83 (69.7%), failure to add a second drug 22 (18.5%), failure to increase dose of a drug 9 (7.6%) and failure to increase frequency of a drug 5 (4.2%) (Fig. 3).

### Predictors of poor glycemic control

On bivariate analysis, age (*p* = 0.013), duration of diabetes (*p* = 0.014), use of herbal medicine (*p* = 0.034), adherence to antidiabetic medication (*p* < 0.001), therapeutic inertia (*p* < 0.001), diabetic knowledge (*p* = 0.026) and type of antidiabetic treatment (*p* = 0.001) were associated with poor glycemic control.

According to the result of multivariate analysis, non-adherence to antidiabetic medication, not having formal education, therapeutic inertia and poor diabetic knowledge were found to be independent predictors of poor glycemic control.

Patients with poor adherence to medication were 3.36 times more likely to have poor glycemic control (AOR = 3.36, 95% CI 1.16–9.72, *p* = 0.04) than patients with good adherence to medication. In addition, patients with no formal education were 3.84 time more likely to have poor glycemic control (AOR = 4.63, 95% CI 1.30–16.43, *p* = 0.018) than patients who attained tertiary education. Moreover, patients with therapeutic inertia were 3.16 times more likely to have poor glycemic control (AOR = 3.16, 95% CI 1.61–6.20, *p* = 0.001) than patients with no therapeutic inertia. Those patients who had poor diabetic knowledge were 4.79 times more likely to have poor glycemic control (AOR = 4.79, 95% CI 1.56–14.68, *p* = 0.006) than patients who had good diabetic knowledge. Therefore, not having formal education, poor adherence to medication, therapeutic inertia and poor diabetic knowledge were found to be independent predictors of poor glycemic control among the study participants (Table 5).

**Table 5:** Multivariate analysis of the associations between poor glycemic control using HbA1C and different covariates of study participants, Jimma Medical Center, Southwest Ethiopia, 2022.

Variable	Glycemic status		COR (95%CI)	*p* value	AOR (95% CI)	*p* value
	Good control	Poor control				
Sex						
Male	59 (37.1)	133 (69.3)	1		1	
Female	27 (23.5)	88 (76.5)	0.69 (0.40–1.17)	0.17	1.11 (0.54–2.28)	0.76
Age (years)						
18–35	24 (38.7)	38 (61.3)	1		1	
36–45	10 (15.4)	55 (84.6)	0.28 (0.12–0.67)	0.004	0.28 (0.06–1.37)	0.11
> 45	52 (28.9)	128 (71.1)	0.64 (0.35–1.17)	0.15	1.03 (0.22–4.76)	0.96
Marital status						
Single	17 (38.6)	27 (61.4)	1		1	
Married	62 (27.7)	162 (72.3)	0.60 (0.31–1.19)	0.14	0.70 (0.15–3.16)	0.64
Divorced	2 (18.2)	9 (81.8)	0.35 (0.06–1.83)	0.21	0.24 (0.02–2.79)	0.26
Widow	5 (17.9)	23 (82.1)	0.34 (0.11–1.08)	0.06	0.17 (0.02–1.25)	0.08
Residence						
Urban	37 (24.7)	113 (75.3)	1		1	
Rural	49 (31.2)	108 (68.8)	1.38 (0.83–2.28)	0.2	1.15 (0.59–2.26)	0.67
Level of education						
No formal education	25 (28.7)	62 (71.3)	1.84 (0.72–4.72)	0.2	3.84 (1.06–13.93)	0.04*
Primary	32 (27.1)	86 (72.9)	1.70 (0.68–4.23)	0.25	1.83 (0.55–6.06)	0.31
Secondary	22 (34.9)	41 (65.1)	2.45 (0.93–6.45)	0.07	1.83 (0.53–6.36)	0.33
Tertiary	7 (17.9)	32 (82.1)	1		1	
Type of DM						
Type 1	20 (35.7)	36 (35.7)	1		1	
Type 2	66 (26.3)	185 (73.7)	0.64 (0.34–1.18)	0.15	1.25 (0.25–6.20)	0.07
Diabetes duration						
< 5 years	52 (26.9)	141 (73.1)	1		1	
5–10 years	25 (33.8)	49 (66.2)	0.79 (0.42–1.48)	0.47	0.64 (0.22–1.87)	0.41
> 10 years	9 (22.5)	31 (77.5)	0.41 (0.22–0.77)	0.006	0.28 (0.09–0.86)	0.05
Number of visits						
6	36 (33.9)	70 (66.1)	1.55 (0.92–2.59)	0.12	1.64 (0.84–3.22)	0.14
12	50 (24.9)	151 (75.1)	1		1	
Use of herbal medicine						
No	79 (26.9)	215 (73.1)	1		1	
Yes	7 (53.8)	6 (46.2)	3.17 (1.03–9.73)	0.04	1.24 (0.16–9.65)	0.83
Therapeutic inertia						
No	86 (45.7)	102 (54.3)	1		1	
Yes	0 (0.0)	119(100%)	4.57 (2.42–8.62)	< 0.001	3.16 (1.61–6.20)	0.001*
Diabetic knowledge						
Poor	63 (33.5)	125 (66.5)	2.30 (0.96–5.51)	0.06	4.79 (1.56–14.68)	0.006*
Acceptable	16 (20)	64 (80)	1.14 (0.42–3.05)	0.79	2.19 (0.63–7.53)	0.21
Good	7 (17.9)	32 (82.1)	1		1	
Type of treatment						
Metformin	7 (14.9)	40 (85.1)	1		1	
Insulin	23 (21.1)	86 (78.9)	1.52 (0.60–3.85)	0.36	1.27 (0.37–4.32)	0.69
Metformin + Glibenclamide	19 (29.7)	54 (70.3)	2.41 (0.91–6.33)	0.07	0.69 (0.19–2.51)	0.58
Insulin + metformin	37 (42.5)	50 (57.5)	4.22 (1.70–10.49)	0.002	0.54 (0.13–2.22)	0.39
Medication adherence						
Poor	33 (14.5)	195 (85.5)	4.09 (1.48–11.31)	< 0.01	3.36 (1.16–9.72)	0.04*
Good	29 (63.0)	17 (37.0)	1		1	

*Significant difference.

## Discussions

The prevalence of poor glycemic control in the present study was 72%. This finding is comparable to studies conducted in Singapore and Yemen where the prevalence of poor glycemic control was 71% and 73.2% respectively^18,19^. Unlike the current study, in studies conducted in Poland (83.9%), Uganda (84.3%), and India (91.8%), magnitude of poor glycemic control was higher^20–22^. This discrepancy may have happened due to the differences in socioeconomic status, environmental factors, clinical characteristics, and lifestyle, which predispose individuals to different risk factors of poor glycemic control. On the contrary, the magnitude of poor glycemic control in the present study was higher than studies conducted in Gondar (60.5%) and Mekelle (61.9%)^23,24^. The reason for the higher prevalence in the present study might be due to differences in study subjects where these studies were conducted only on type 2 diabetes patients.

In the present study, the mean HbA1C was 8.97 ± 2.74. This value is comparable to studies conducted in Bangladesh and China where mean HbA1C was 9.1 ± 2.54 and 9.0% ± 2.2% respectively^25,26^. In contrast, the mean HbA1C value in the present study was higher than studies conducted in Gondar (7.82 ± 19) and Jimma Medical Center (7.6 ± 1.9)^14,27^. This finding highlights the need for collaboration between health professionals in reducing the rate of uncontrolled diabetes.

This study found that 61.2% of the patients had poor knowledge about diabetes. This finding is lower than the finding from the study conducted in Addis Ababa where 80.3% of the patients had inadequate knowledge about diabetes^28^. In contrast, the finding in the present study is higher than a study conducted in Jimma where 30% of the patients had inadequate knowledge^29^. This variation could be related to a difference in the scoring and categorization of knowledge question items; where this study used a mean score of knowledge item questions to categorize respondents in to adequate and inadequate knowledge level whereas a study conducted in Jimma used 60% score and above as satisfactory knowledge level.

The results of the present study indicated that diabetic patients with no formal education had a higher risk of poor glycemic control when compared to those having a high level of education. This finding is consistent with studies conducted in Yemen, Nigeria and Jimma, Shenen Gibe hospital^19,30,31^. A high level of education, on the other hand, can allow a patient to acquire special skills related to problem-solving and may enhance his/her ability to cope with the disease, manage it and better control his/her blood glucose levels^30^.

Majority of patients in this study were non-adherent to their antidiabetic medications. Non-adherence was an independent predictor of poor glycemic control. Similar to this finding, a study conducted in Jimma Shenen gibe hospital described good medication adherence as a predictor of good glycemic control^31^. Poor glycemic control was higher among non-adherent patients in the current study. This finding is consistent with other studies conducted in Turkey and Jordan^32,33^. Literature shows that low treatment adherence continues to be a considerable barrier that prevents many diabetic patients from achieving good glycemic control. Increased compliance is associated with substantial improvements in glycemic control in health care^29^.

Therapeutic inertia was another predictor of poor glycemic control as per the current study and was observed in 53.8% of diabetic patients with poor glycemic control. This finding is in line with a study conducted in Canada where 55% of diabetic patients had therapeutic inertia^12^. On the other hand, the finding of the present study was higher than a study carried out in United Kingdom. The prevalence of therapeutic inertia in the UK study was 26%^11^. The reason for the higher rate of therapeutic inertia in the present study might be related to the lack of diabetes specialists and failure of physicians to practice as per treatment guidelines.

## Conclusion

Nearly three fourth of diabetic patients had poor glycemic control. Predictors of poor glycemic control were non-adherence to medication, poor diabetic knowledge, no formal education, and therapeutic inertia. Therefore, health care providers of JMC should work in collaboration on therapeutic decision making to reduce impact of therapeutic inertia and clinical pharmacists and nurses should take part in educating patients about medication adherence and diabetic knowledge.

## Data Availability

The datasets used and/or analyzed during the current study can be available from the corresponding author on reasonable request.

## Abbreviations

ADA: American Diabetes Association
DM: Diabetes mellitus
HbA1C: Glycated hemoglobin A1C
JMC: Jimma Medical Center
TI: Therapeutic inertia
T1DM: Type one diabetes mellitus
T2DM: Type 2 diabetes mellitus
